# HOTAIR is a therapeutic target in glioblastoma

**DOI:** 10.18632/oncotarget.3229

**Published:** 2015-03-21

**Authors:** Xuan Zhou, Yu Ren, Jing Zhang, Chuanbao Zhang, Kailiang Zhang, Lei Han, Lingping Kong, Jianwei Wei, Luyue Chen, Jingxuan Yang, Qixue Wang, Jianning Zhang, Yuqi Yang, Tao Jiang, Min Li, Chunsheng Kang

**Affiliations:** ^1^ Department of Neurosurgery, Tianjin Medical University General Hospital; Laboratory of Neuro-oncology, Tianjin Neurological Institute, Tianjin 300052, China; ^2^ The Department of Otorhinolaryngology and Maxillofacial Oncology, Tianjin Medical University Cancer Institute & Hospital; Key Laboratory of Cancer Prevention and Therapy, Tianjin Cancer Institute; National Clinical Research Center of Cancer, Tianjin 300060, China; ^3^ Tianjin Research Center of Basic Medical Science, Tianjin Medical University, Tianjin 300070, China; ^4^ Beijing Institute of Genomics, Chinese Academy of Sciences, Beijing 100101, China; ^5^ Beijing Neurosurgical Institute, Department of Neurosurgery, Beijing Tiantan Hospital, Capital Medical University, Beijing 100050, China; ^6^ Department of Medicine, Department of Surgery, The University of Oklahoma Health Sciences Center, Oklahoma City, OK 73104, USA; ^7^ Department of Pharmacology, Tianjin Medical University, Tianjin 300070, China

**Keywords:** HOTAIR, NLK (Nemo-like kinase), β-catenin, PRC2 (Polycomb repressive complex 2), Glioblastoma

## Abstract

HOTAIR is a negative prognostic factor and is overexpressed in multiple human cancers including glioblastoma multiform (GBM). Survival analysis of Chinese Glioma Genome Atlas (CGGA) patient data indicated that high HOTAIR expression was associated with poor outcome in GBM patients. NLK (Nemo-like kinase), a negative regulator of the β-catenin pathway, was negatively correlated with HOTAIR expression. When the β-catenin pathway was inhibited, GBM cells became susceptible to cell cycle arrest and inhibition of invasion. Introduction of the HOTAIR 5′ domain in human glioma-derived astrocytoma induced β-catenin. An intracranial animal model was used to confirm that HOTAIR depletion inhibited GBM cell migration/invasion. In the orthotopic model, HOTAIR was required for GBM formation *in vivo*. In summary, HOTAIR is a potential therapeutic target in GBM.

## INTRODUCTION

Glioblastoma multiform (GBM) is the most malignant tumor of the human central nervous system. Despite advances in combination treatments of radiation and chemotherapy following surgical resection of the tumor, the prognosis of GBM remains poor, with an average survival time of less than one year [1, 2].

The epidermal growth factor receptor (EGFR) is overexpressed in a variety of human epithelial tumors [3]. Amplification of the EGFR gene, with the subsequent overexpression of the EGFR protein, is the most common genetic alteration in GBM, occurring at a frequency of approximately 34–63% [4, 5]. Aberrant signaling of EGFRvIII (the most common extracellular mutation) has been shown to be important in driving tumor progression and often correlates with poor prognosis for GBM patients [6, 7].

Mounting evidence has demonstrated that a large number of noncoding RNAs (ncRNAs) are involved in human cancers, including glioma [8, 9]. Up to 3000 human long noncoding RNAs (lncRNAs) have been shown to have important functions in human normal or disease states [10]. HOTAIR is a ~2000bp lncRNA that is encoded antisense to the HOXC locus, one of the four chromosomal loci (HOXA to D) containing the clustered HOX genes [8, 11]. HOTAIR regulates the transcriptional silencing of genes of the HOXD locus and other genetic loci by binding to Polycomb repressive complex 2 (PRC2) (EZH2, SUZ12 and EED) at its 5′ end and localizing it to the specific site where H3K27 trimethylation and epigenetic silencing of gene expression occur [10]. Moreover, HOTAIR interacts with another chromatin complex, the lysine-specific demethylase 1 (LSD1)-CoREST complex, which mediates the removal of mono- and di-methylation of H3K4, a histone mark associated with gene activation, in nucleosomes [11–13].

Current research indicates that HOTAIR is a negative prognostic factor for survival of patients with breast and colon cancer and glioma, and increased HOTAIR expression in patients has been correlated with increased metastasis [11, 14–16]. Our previous research indicated that HOTAIR promoted malignant progression and poor prognosis in glioma patients and exhibited pro-oncogenic activity [16].

Abnormal activation of the Wnt/β-catenin pathway has been reported to participate in malignant progression and has been implicated in poor prognosis of malignant glioma cells, highlighting it as a potential therapeutic target for gliomas [17–19]. Canonical Wnt signaling activates target genes by promoting association of the co-activator β-catenin with TCF/LEF transcription factors [20]. NLK phosphorylates TCF/LEF and inhibits the interaction of the β-catenin-TCF complex with the DNA binding region of downstream targets [21].

However, the therapeutic potential of HOTAIR in GBM is not well defined, and the underlying mechanisms are unknown. Therefore, in the present study, we aimed to determine the role of HOTAIR in regulating the β-catenin pathway. We determined that GBM cell cycle progression and invasion were significantly attenuated when HOTAIR was depleted.

## RESULTS

### High expression of HOTAIR correlates with NLK expression and confers a poor prognosis in GBM patients

**Figure 1:**
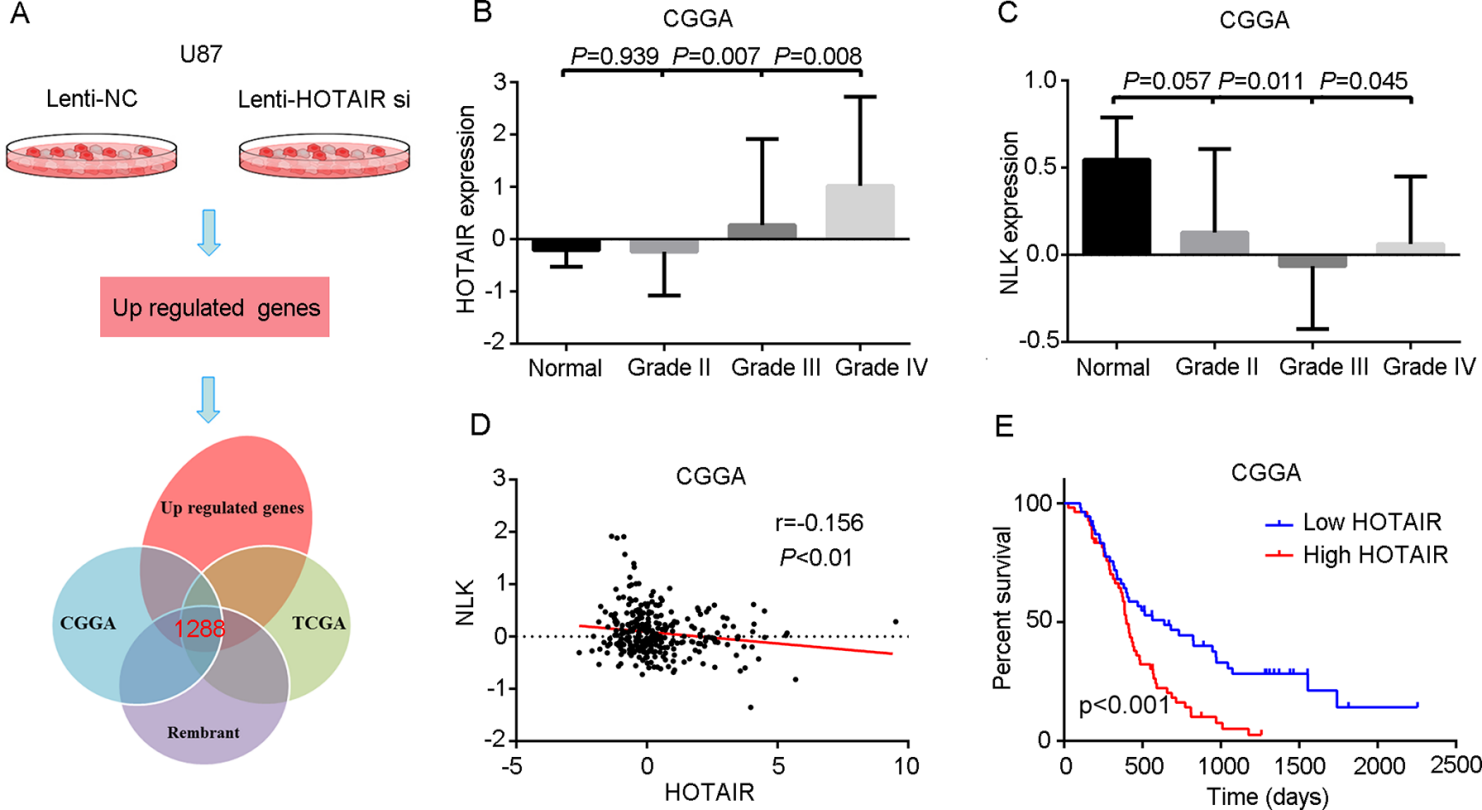
High levels of HOTAIR correlate with NLK expression and confer a poor prognosis in GBM patients **(A)** The sequencing data from HOTAIR-depleted U87 cells overlapped with that of glioma genome atlas public databases. **(B)** HOTAIR was highly expressed in grade IV gliomas (*P* < 0.05). **(C)** NLK was expressed at a lower level in grade III and IV gliomas (*P* < 0.05). **(D)** Pearson's correlation analysis indicated that HOTAIR expression was negatively associated with NLK expression (*r* = 0.156, *P* < 0.01). **(E)** Kaplan–Meier survival curve analysis indicated that GBM patients with lower HOTAIR expression showed prolonged survival compared with patients with high levels of HOTAIR (*P* < 0.001).

First, we analyzed changes in mRNA levels in HOTAIR-depleted U87 GBM cells. In total, we validated differential expression of 1,288 upregulated genes and 1,628 downregulated genes compared with cells treated with Lenti-NC virus (Figure 1A). Furthermore, we combined the mRNA sequencing data and the publically available microarray gene expression data for glioblastoma patients: 123 samples of GBM from TCGA (Agilent 4502A-1) [22], 34 samples of GBM from CGGA2 [23], 227 samples of GBM from Rembrandt [24], 79 samples of GBM from TTseq [25], and 77 samples of GBM from GSE4290 [26]. For each data cohort, Pearson correlation was used to evaluate the correlation between the expression of HOTAIR and that of other genes. A significant HOTAIR-associated gene was identified when the *P* value of the correlation was less than 0.05. We discovered that the expression of NLK was significantly associated with that of HOTAIR in four data cohorts except in TTseq data cohort (see Supplementary Figure S1A–S1E).

Second, we measured HOTAIR expression by whole gene profiling of 310 glioma and 5 normal brain tissues (CGGA1). The level of HOTAIR expression in GBM was higher than in other low grade gliomas and normal brain tissues (*P* < 0.05, Figure 1B). In addition, NLK was decreased in grade III and IV gliomas compared with grade II gliomas and normal brain tissues (*P* < 0.05, Figure 1C). Pearson's correlation analysis indicated that HOTAIR expression was negatively associated with NLK expression in 108 GBM samples of the cohort (*r* = 0.156, *P* < 0.05; Figure 1D). Kaplan-Meier survival analysis showed that patients with low HOTAIR expression (*n* = 54) had significantly increased overall survival compared with patients with high HOTAIR expression (*n* = 54, *P* < 0.05; Figure 1E).

Third, a high level of HOTAIR expression was associated with age at diagnosis (*P* = 0.009), MGMT promoter methylation (*P* < 0.05, Table 1) in all the analyzed GBM samples. Gender, KPS score, resection status, and expression of MGMT, Ki-67, and PTEN were not correlated with HOTAIR expression. Next, we conducted univariate Cox regression analysis using clinical and genetic variables for 109 primary GBM patients from the CGGA1 cohort and found that high expression of HOTAIR, age at diagnosis, IDH1 mutation, KPS score, and Ki-67 expression were statistically associated with overall survival. Then we evaluated these factors with *p* value < 0.05 by using a multivariate Cox proportional hazards model. The analysis revealed that HOTAIR expression, age at diagnosis, KPS score, and Ki-67 expression correlated independently with overall survival (Table 2).

**Table 1:** HOTAIR expression was associated with clinical and molecular pathology features in a cohort of 108 GBM samples

Variable	HOTAIR Low	HOTAIR High*	*p* value
Gender (F/M)	26/28	16/39	0.041
Age at diagnosis (years)	44.65 ± 12.51	50.84 ± 11.83	0.009
Overall survival (days)	639	398	< 0.001
KPS (≥ 80/< 80)	30/24	27/27	0.563
Resection (total/subtotal)	22/32	21/33	0.844
IDH1 mutation (N/Y)	43/11	53/2	0.020
MGMT promoter methylation (U/M/ND)	21/22/11	40/10/5	0.002
MGMT (L/H/ND)	20/34/0	19/31/5	0.076
Ki-67 (L/H/ND)	17/37/0	18/32/5	0.068
EGFR (L/H/ND)	27/27/0	13/37/5	0.013
PTEN (L/H/ND)	2/51/1	6/44/5	0.075

ND, not determined

* one sample lost at follow up

**Table 2:** Cox Hazards Regression Analyses of Clinicopathologic Factors and HOTAIR expression in a cohort of 108 GBM samples

Variable	Univariate Cox Regression			Multivariate Cox Regression		
	HR	95% CI	*p* value	HR	95% CI	*p* value
HOTAIR	1.171	1.056–1.299	0.003	1.149	1.008–1.309	0.037
Gender	1.129	0.732–1.741	0.583			
Age at diagnosis	1.026	1.007–1.045	0.007	1.021	1.000–1.042	0.052
IDH1 mutation	0.436	0.216–0.879	0.020	0.553	0.263–1.163	0.118
MGMT promoter methylation	0.689	0.420–1.129	0.139			
KPS	0.975	0.959–0.991	0.002	0.970	0.954–0.987	0.001
Resection	1.430	0.924–2.214	0.108			
MGMT	0.963	0.789–1.174	0.707			
PTEN	0.785	0.578–1.066	0.121			
EGFR	1.091	0.922–1.291	0.308			
Ki–67	1.538	1.186–1.995	0.001	1.385	1.058–1.813	0.018

### HOTAIR regulates the β-catenin signaling pathway by inhibiting the transcription of NLK

**Figure 2:**
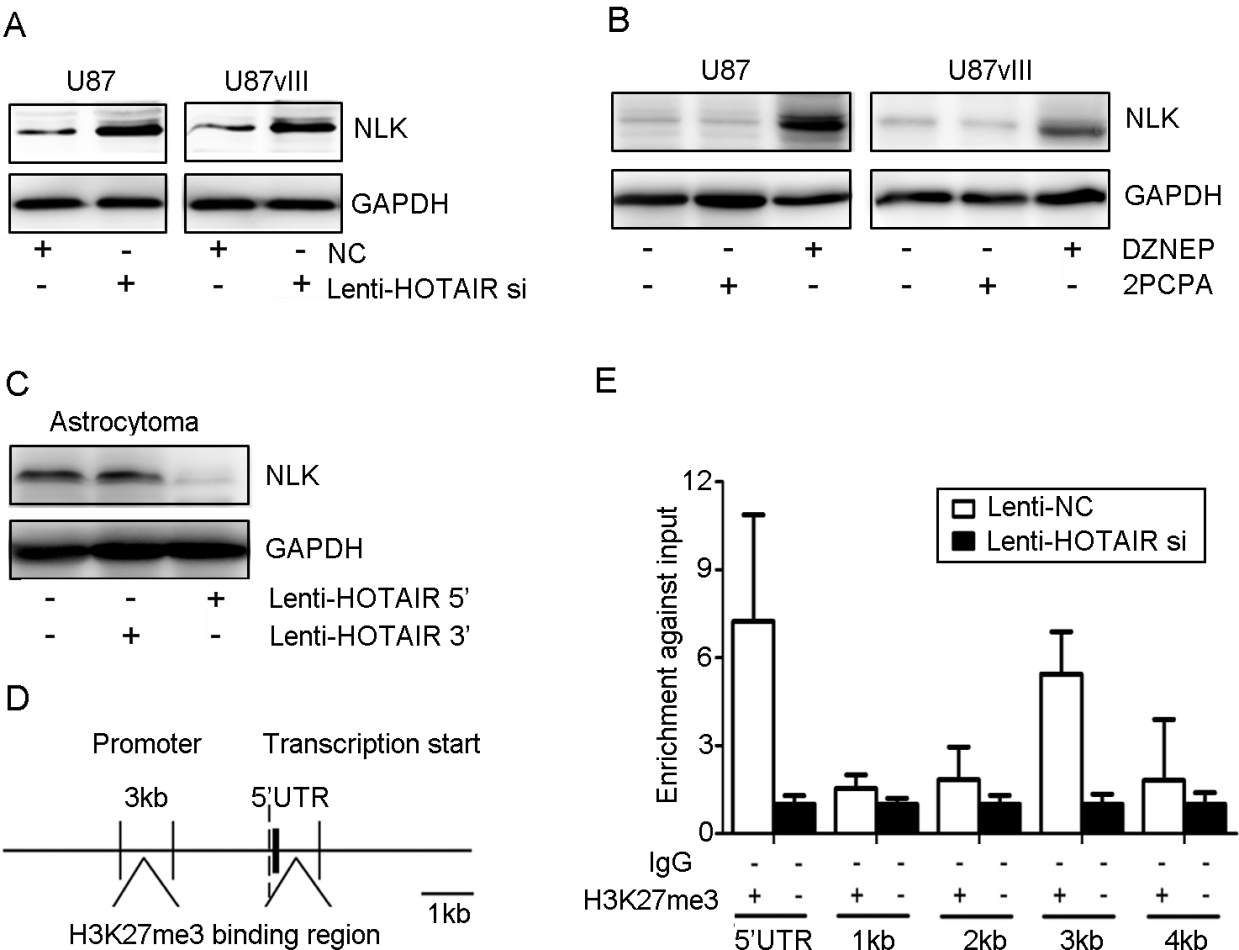
HOTAIR inhibited NLK transcription *in vitro* **(A)** Lenti-HOTAIR si treatment induced NLK expression in U87 and U87vIII GBM cell lines after 48 h. **(B)** U87 and U87vIII GBM cell lines were incubated with 5 μmol/L DZNEP or 2PCPA for 48 h, and DZNEP treatment increased the level of NLK. **(C)** Astrocytoma cells were treated with Lenti-HOTAIR 3′ or 5′ domain for 48 h, and treatment with Lenti-HOTAIR 5′ domain inhibited NLK expression. GAPDH was used as a loading control. **(D)** and **(E)** The interaction of H3K27me3 and NLK-encoding gene regulatory elements (an approximately 5 kb region upstream from the transcriptional start site) was determined by CHIP assay.

HOTAIR knockdown induces or represses multiple genes that could contribute to the functional pro-oncogenic activity of HOTAIR in GBM. Therefore, we measured NLK expression in Lenti-HOTAIR si-treated GBM cells and found that NLK expression was significantly elevated compared with the Lenti-NC-treated cells (Figure 2A). DZNEP and 2PCPA, an EZH2 or LSD1 inhibitor that may block the function of the HOTAIR 5′ or 3′ domain, was used to further study the role of HOTAIR in regulating target gene expression. DZNEP treatment increased the expression of NLK in both U87 and U87vIII GBM cells (Figure 2B). 2PCPA treated U87 and U87vIII GBM cells showed no significant NLK expression (Figure 2B). Interestingly, introduction of the HOTAIR 5′ domain into an astrocytoma-derived primary culture dramatically decreased NLK expression, whereas the HOTAIR 3′ domain did not have this effect (Figure 2C).

To study the underlying mechanism by which NLK transcription was suppressed by HOTAIR-mediated H3K27 trimethylation, we analyzed the promoter and 5′ UTR region of the NLK gene (approximately 5,000 bp) using an H3K27me3 CHIP assay. CHIP-PCR results indicated that HOTAIR knockdown inhibited H3K27me3 binding to NLK gene promoter regulatory elements (Figure 2D and 2E).

**Figure 3:**
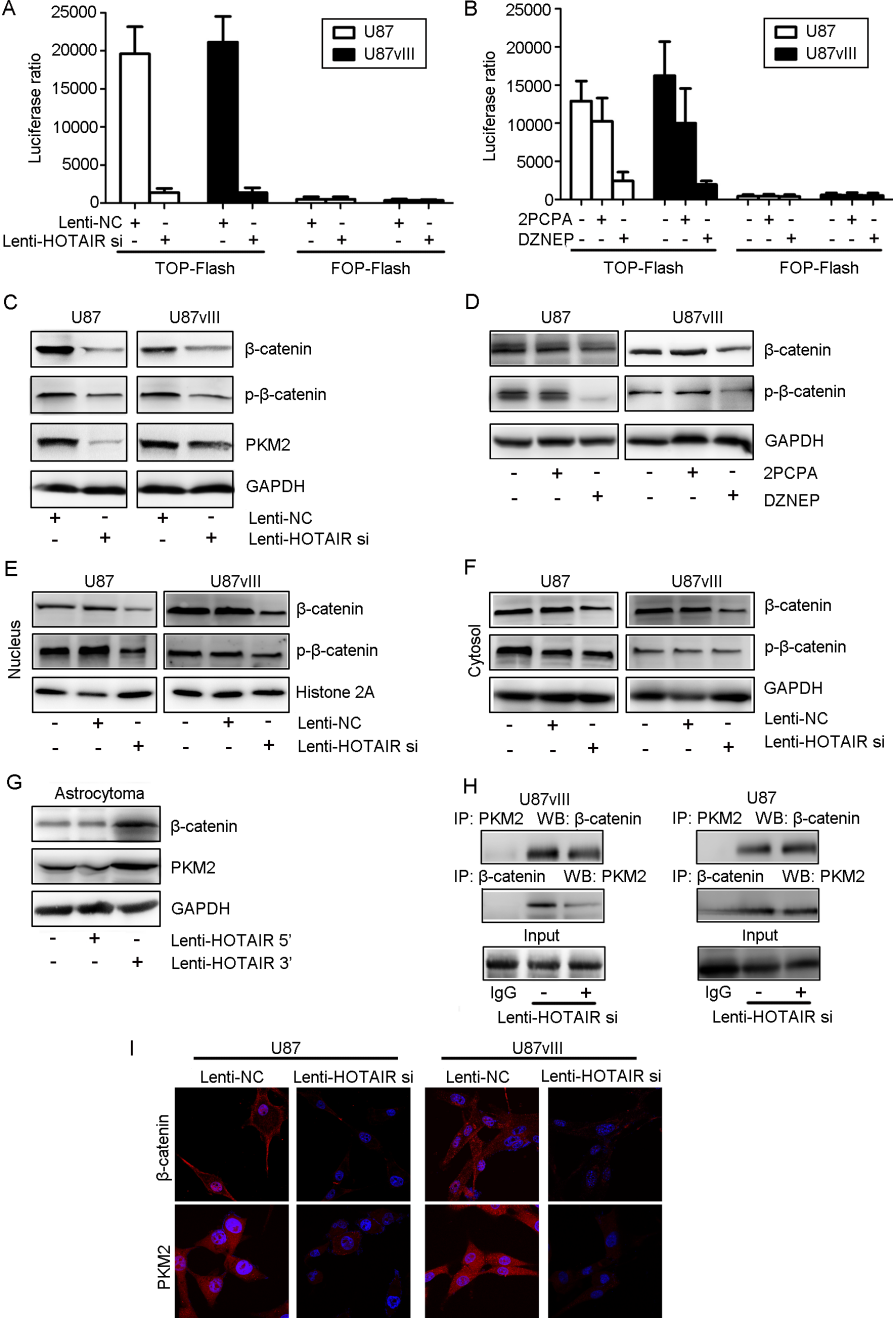
HOTAIR regulated the activity of the β-catenin signaling pathway *in vitro* **(A)** A TOP flash reporter assay indicated that Lenti-HOTAIR si treatment inhibited β-catenin transcriptional activity (*P* < 0.01). **(B)** A TOP flash reporter assay indicated that DZENP treatment inhibited β-catenin transcriptional activity to a greater degree than 2PCPA treatment (*P* < 0.05). **(C)** Lenti-HOTAIR si treatment inhibited β-catenin and PKM2 expression and decreased p-β-catenin levels in whole cell lysates of U87 and U87vIII cells. **(D)** DZENP treatment inhibited the expression of β-catenin and decreased p-β-catenin levels in whole cell lysates of U87 and U87vIII cells. **(E)** Lenti-HOTAIR si treatment inhibited β-catenin expression and decreased p-β-catenin levels in the nucleus lysate in U87 and U87vIII cells. **(F)** Lenti-HOTAIR si treatment inhibited β-catenin expression and decreased p-β-catenin levels in the cytosol lysate in U87 and U87vIII cells. **(G)** Treatment with the Lenti-HOTAIR 5′ domain increased the levels of β-catenin and PKM2. **(H)** Freshly isolated cell lysates (U87 and U87vIII cells infected with Lenti-HOTAIR si or Lenti-NC) were used to immunoprecipitate β-catenin or PKM2 with specific antibodies. Whole immunoglobulin (IgG) was used as a control antibody for immunoprecipitation assays. The immunoprecipitated complexes were subjected to Western blot analysis with specific antibodies against β-catenin and PKM2 as indicated. GAPDH or Histone 2A was used as a loading control. **(I)** Compared with Lenti-HOTAIR si-treated cells, Lenti-NC-treated cells exhibited higher β-catenin and PKM2 expression in both the cytoplasm and the nucleus (magnification: 1000x).

Previous studies indicated that NLK inhibits the β-catenin signaling pathway in glioma. Thus, we next measured whether HOTAIR deletion affect β-catenin transcriptional activity by the TOP/FOP flash reporter plasmid. Compared with Lenti-NC-treated cells, remarkably decreased activity of the β-catenin/TCF4 pathway in Lenti-HOTAIR si-treated cells was detected in both U87 and U87vIII GBM cells (*P* < 0.05, Figure 3A). DZNEP treatment, rather than 2PCPA, significantly inhibited β-catenin/TCF4 activity by TOP/FOP flash reporter assay (Figure 3B). Western blotting results showed that the Lenti-HOTAIR si plasmid decreased the expression of β-catenin and the levels of p-β-catenin, particularly in whole cell lysate, nucleus and cytosol lysate (Figure 3C, 3E and 3F). More importantly, in DZNEP-treated cells, the levels of β-catenin and p-β-catenin in the nucleus were decreased (Figure 3D). Introducing HOTAIR 5′ domain remarkably increased NLK expression in astrocytoma while introducing HOTAIR 3′ domain didn't (Figure 3G). Nuclear recruitment of PKM2 was required for nuclear accumulation of β-catenin, and in HOTAIR inhibited U87 and U87vIII cells, both IP (Immunoprecipitation) and IF (Immunofluorescence) results indicated that nuclear PKM2 expression was significantly suppressed (Figure 3H, 3I).

### HOTAIR regulates GBM cell cycle progression *in vitro*

**Figure 4:**
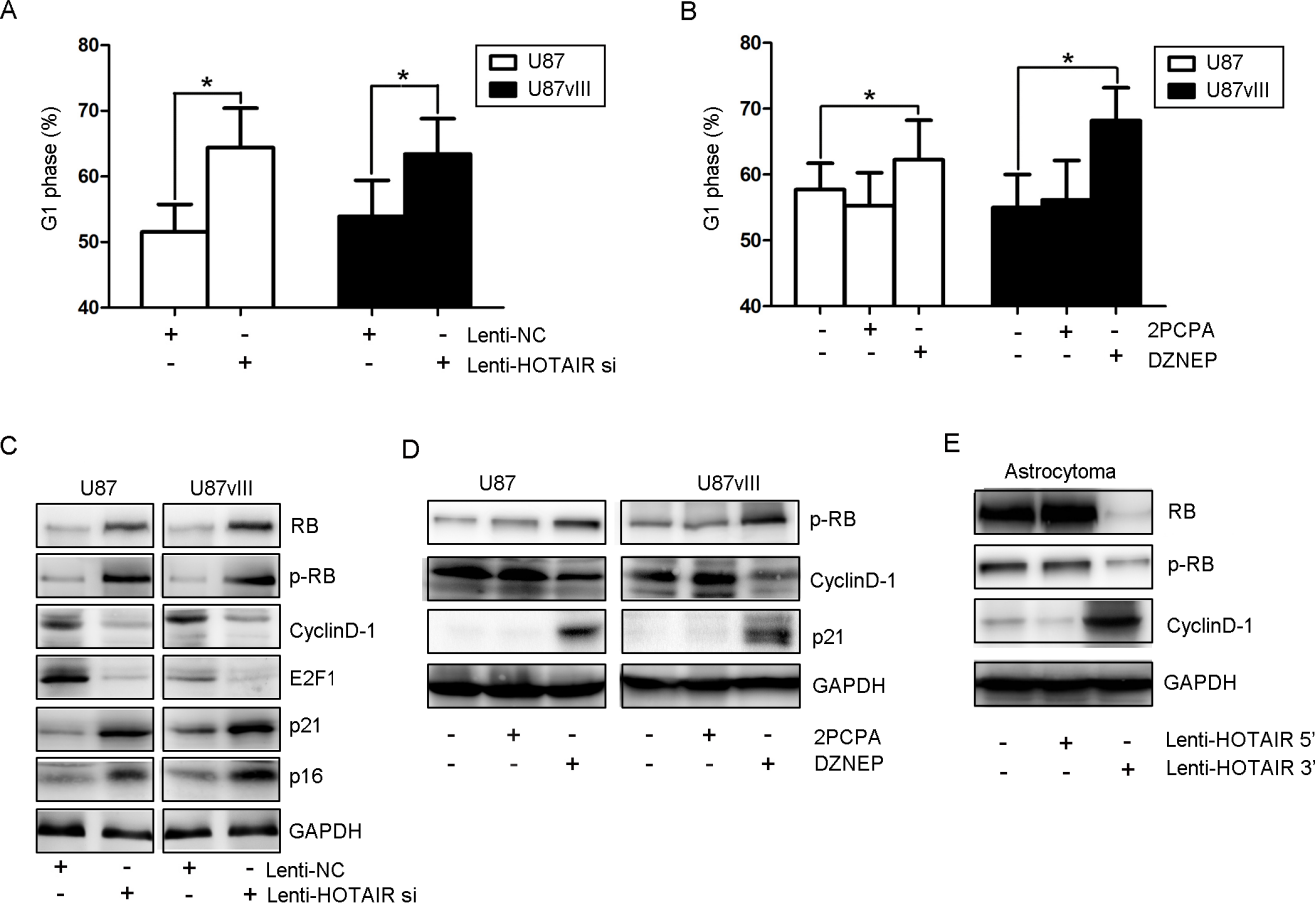
HOTAIR regulates GBM cell cycle progression *in vitro* **(A)** The cell cycle distribution of U87 and U87vIII cells treated with Lenti-HOTAIR si and Lenti-NC was determined by FCM. **(B)** The cell cycle distribution of U87 and U87vIII cells treated with DZNEP and 2PCPA was determined by FCM. **(C)** Lenti-HOTAIR si treatment increased the expression of RB, p21 and p16 and the levels of p-RB, whereas the expression of cyclin D1 and E2F1 was inhibited in U87 and U87vIII cells. **(D)** DZNEP treatment induced the expression of p21 and increased the levels of p-RB while inhibiting cyclin D1 expression in U87 and U87vIII cells. **(E)** Treatment with Lenti-HOTAIR 5′ domain inhibited the expression of RB and decreased levels of p-RB while inducing the expression of cyclin D1 in an astrocytoma primary culture. GAPDH was used as a loading control.

Our previous report indicated that HOTAIR knockdown blocked the cell cycle at G1 phase [16]. To further study the underlying mechanism of this phenomenon, we compared the cell cycle distribution after treatment with Lenti-HOTAIR si and the EZH2 inhibitor for 48 hr. Flow cytometry data revealed that both HOTAIR-depleted U87 and U87vIII GBM cells increased about 1.3–1.5 fold in G1 phase and a decreased 1.4–1.7 fold in replicating S phase (Figure 4A, *P* < 0.05). The tumor suppressor retinoblastoma protein (RB) and the CDK inhibitors p21 and p16 were upregulated upon HOTAIR knockdown. Notably, HOTAIR-depleted cells also showed an increased level of phosphorylated RB (Figure 4C). Cell cycle arrest phenotypes, including G1 arrest, and a significant reduction in the percentage of cells in S phase were also observed in DZNEP-treated U87 and U87vIII cells, whereas 2PCPA treatment did not affect the cell cycle (Figure 4B and 4D). Moreover, we overexpressed the HOTAIR 3′ or 5′ domain in an astrocytoma-derived primary culture (Figure 4E). Western blots indicated that overexpression of the 5′ domain decreased the levels of pRB and upregulated cyclinD1 expression. These data provide important evidence that the HOTAIR 5′ domain binds to EZH2 in GBM cells and regulates cell cycle progression.

### HOTAIR regulates GBM invasion *in vitro*

**Figure 5:**
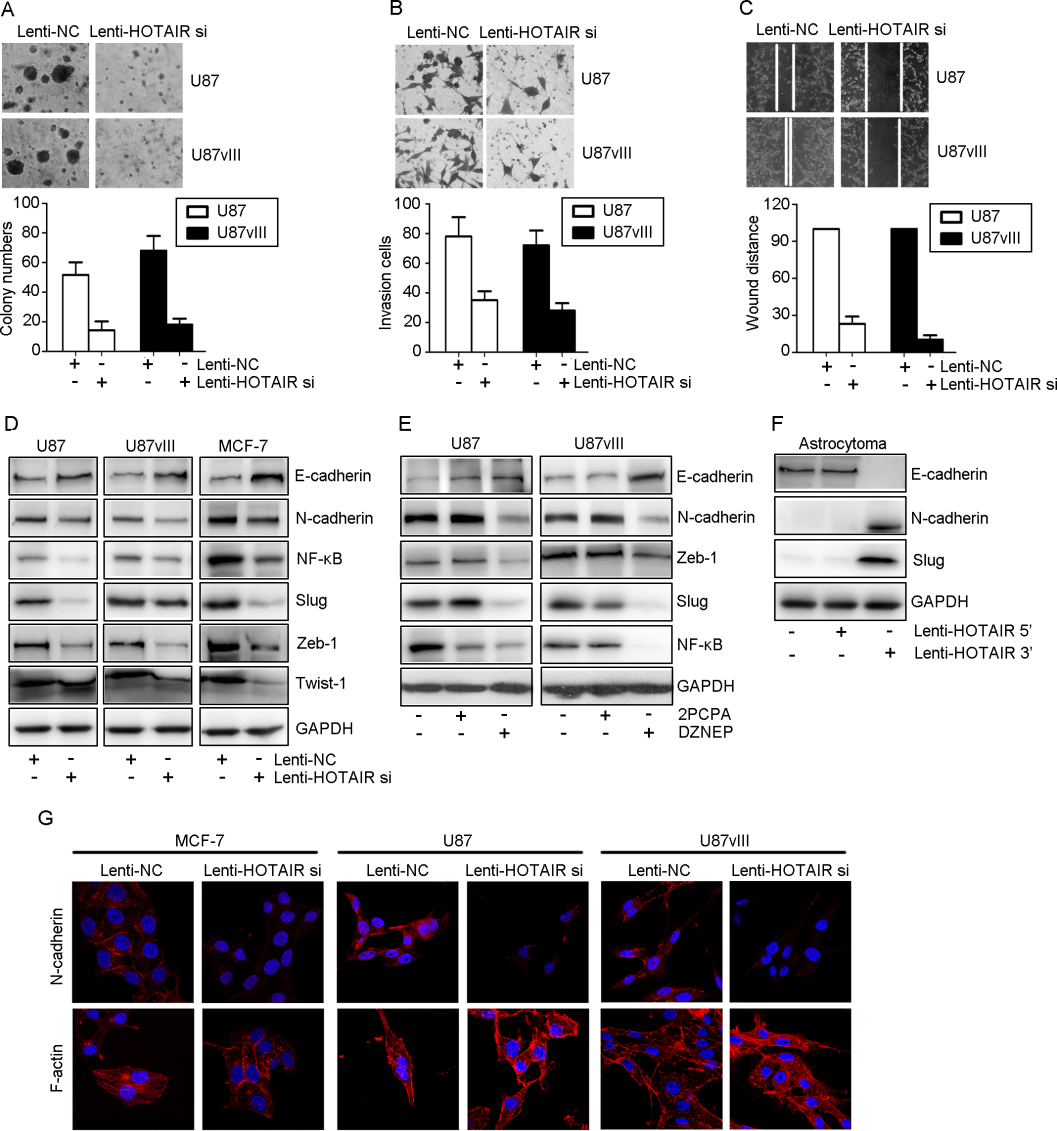
HOTAIR regulated GBM invasion *in vitro* **(A)** A 3D matrigel assay indicated that Lenti-HOTAIR si inhibited proliferation and the ability to dehydrate ECM in U87 and U87vIII cells (*P* < 0.05). **(B)** Transwell assays indicated that Lenti-HOTAIR si inhibited invasion of U87 and U87vIII cells (*P* < 0.05). **(C)** Wound healing assays indicated that Lenti-HOTAIR si inhibited cell migration in U87 and U87vIII cells (*P* < 0.05). **(D)** Lenti-HOTAIR si treatment induced E-cadherin expression and inhibited the expression of N-cadherin, NF-κB, Slug, Zeb-1 and Twist-1 in MCF-7, U87 and U87vIII cells. **(E)** DZNEP treatment induced E-cadherin expression and inhibited N-cadherin, NF-κB, Zeb-1 and Slug expression in U87 and U87vIII cells. **(F)** Treatment with Lenti-HOTAIR 5′ domain inhibited E-cadherin expression while inducing N-cadherin and Slug expression in an astrocytoma primary culture. GAPDH was used as a loading control. **(G)** Immunofluorescence staining showed that treatment with Lenti-HOTAIR si inhibited cytoplasmic N-cadherin levels in MCF-7, U87 and U87vIII cells. F-actin staining showed a stress-fiber pattern in Lenti-NC-treated cells and a cortical pattern in Lenti-HOTAIR si-treated cells (magnification: 1000x).

HOTAIR has been characterized as a molecule involved in cancer cell invasion. Consistent with these findings, our research revealed that HOTAIR is involved in GBM cell ECM function. Consequently, we determined HOTAIR regulates the ability of GBM cells to migrate and invade by evaluating EMT-related markers, including E/N-cadherin and etc. The clone diameter of HOTAIR depleted U87 and U87vIII cell was smaller than the Lenti-NC treated cells (Figure 5A). Lenti-HOTAIR si treatment decreased the number of both U87 and U87vIII cells that migrated through the Transwell membrane by 50% compared with Lenti-NC treated cells (Figure 5B). Inhibition of HOTAIR decreased U87 and U87vIII cell migration, as shown by wound healing assays (Figure 5C). In addition, Lenti-HOTAIR si treatment increased E-cadherin expression by 2-fold and decreased N-cadherin at the protein level (Figure 5D). Accordingly, DZNEP treatment induced similar alterations in E/N-cadherin, Zeb-1, Slug and NF-κB (Figure 5E). In contrast, 2-PCPA did not affect EMT markers. We also employed a gain-of-function method to further analyze the role of HOTAIR in GBM cell invasion. Expression of the 5′ domain of HOTAIR introduced into astrocytoma cells induced N-cadherin and Slug expression and suppressed E-cadherin expression. However, expression of the HOTAIR 3′ domain did not affect the expression of EMT markers in GBM cells (Figure 5F).

We next performed immunofluorescence staining to evaluate N-cadherin expression and F-actin morphological changes induced by HOTAIR depletion. In order to prove these changes were universal, we employed a breast cancer cell line (MCF-7) for research use. As shown in Figure 5G, in all cell lines, Lenti-HOTAIR si-infected cells showed epithelial cell features and were characterized by decreased N-cadherin expression. Moreover, F-actin showed a cortical pattern that is typical of epithelial cells. In contrast, the cells treated with Lenti-NC showed a mesenchymal morphology, as indicated by N-cadherin expression in the cytoplasm. Morphologically, F-actin showed a stress fiber pattern.

E- and N-cadherin are core proteins that determine epithelial cell phenotype because lack of expression in cancer cells drives the EMT process and promotes cancer cell migration or invasion [27]. E-cadherin was reported to cross-talk with ZEB1 [28], NF-κB [29] and twist1 [30], and we validated that Lenti-HOTAIR si treatment led to a decrease in the expression of ZEB1, NF-κB and twist1 in GBM and MCF-7 cells. These results confirmed that HOTAIR plays a crucial role in regulating EMT by affecting GBM cell invasion and migration.

### HOTAIR knockdown inhibited tumor growth of an *in vivo* U87 and U87vIII orthotopic GBM model

**Figure 6:**
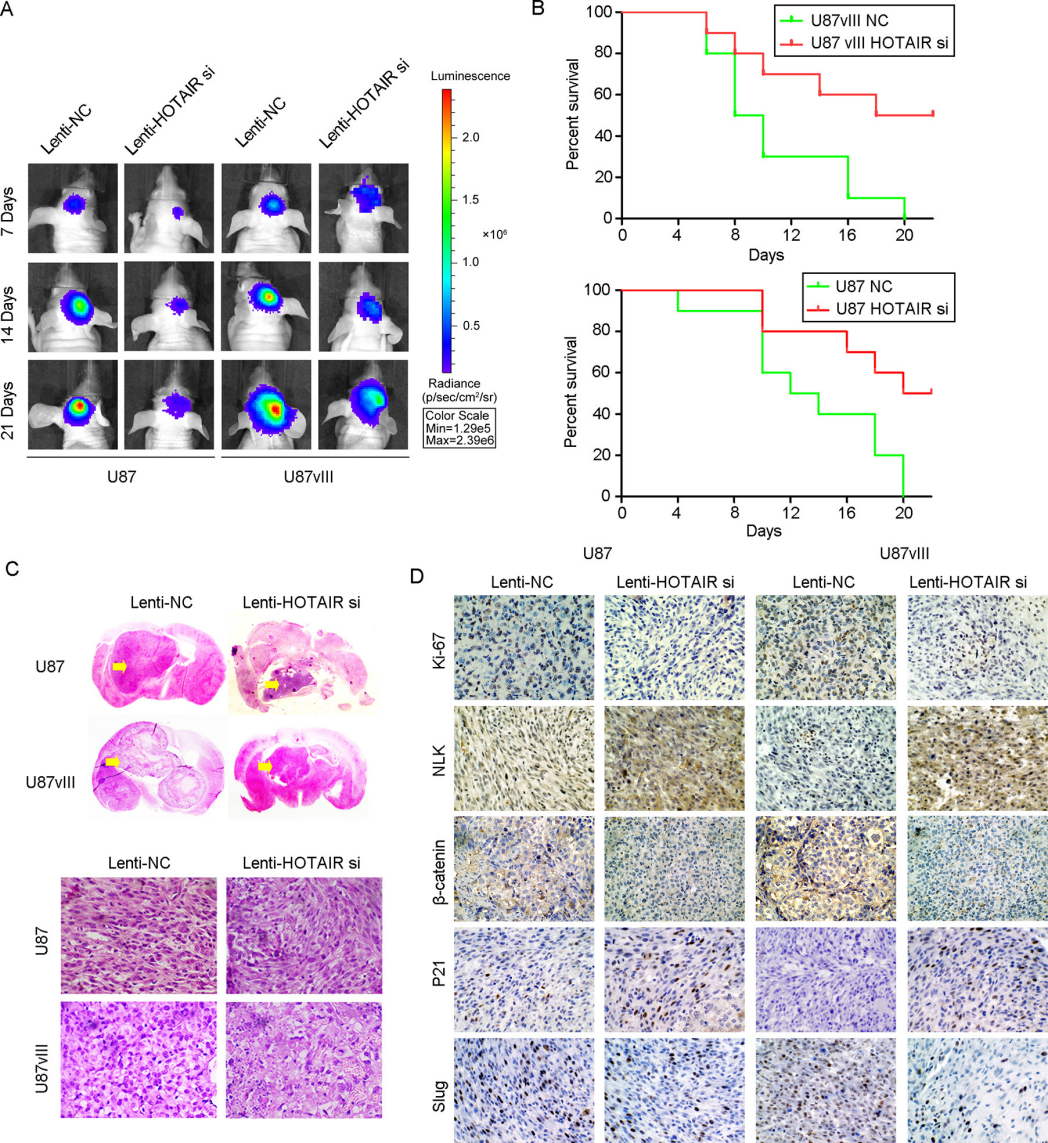
HOTAIR knockdown inhibited tumor growth in a U87 and U87vIII orthotopic GBM model *in vivo* **(A)** Tumor volume of Lenti-NC- or Lenti-HOTAIR si-treated animals at 7, 14 and 21 days after tumor implantation was determined by a bioluminescence imaging system. **(B)** The survival of mice with tumors derived from Lenti-NC- or Lenti-HOTAIR si-treated cells as demonstrated by Kaplan-Meier survival curves. **(C)** Representative images of HE staining of tissue from mice with orthotopic tumors derived from Lenti-NC- or Lenti-HOTAIR si-treated U87 and U87vIII cells (magnification: 200x). **(D)** Representative images of immunohistochemical staining of Ki67, NLK, β-catenin, p21 and Slug in tissue from mice with orthotopic tumors derived from U87 and U87vIII cells treated with Lenti-NC or Lenti-HOTAIR si (magnification: 200x).

To further verify the role of HOTAIR and to determine the therapeutic potential of depleting HOTAIR, we established GBM orthotopic mouse models using U87 and U87vIII cell lines as described previously [31]. For both models, Lenti-HOTAIR si resulted in a significant reduction of the intracranial tumor volume compared with the Lenti-NC groups (Figure 6A). Compared with nude mice injected with Lenti-NC-treated U87 cells (median survival: 9 days) and U87vIII cells (median survival: 13 days), the mice bearing tumors derived from HOTAIR-depleted U87 cells (median survival: 20 days) and U87vIII cells (median survival: 21 days) showed prolonged survival by Kaplan-Meier survival curves (*P* < 0.05; Figure 6B). *In vivo* bioluminescence imagingindicated that the volume of tumors derived from HOTAIR-depleted cells was decreased compared with tumors derived from control cells in both cell lines (80% reduction in U87 with *p* < 0.05 and 75% reduction in U87vIII with *p* < 0.05; Figure 6A). Morphologically, HE-stained slides indicated that HOTAIR knockdown induced shrinkage of tumor cell nuclei (Figure 6C). We next performed IHC staining to evaluate the pathological changes in each of the GBM orthotopic tumors. After HOTAIR knockdown, both U87 and U87vIII GBM cells showed significant changes in the β-catenin pathway, cell cycle regulators and EMT markers. For each model, representative sections are shown in Figure 6D. Compared with control tumors, Lenti-HOTAIR si treatment significantly inhibited β-catenin and Ki-67 expression, and elevated NLK expression. For cell cycle regulators, the expression of p21 was upregulated, indicating that Lenti-HOTAIR si treatment block the cell cycle of GBM cells at G1 phase *in vivo*. For EMT markers, Lenti-HOTAIR si-treated tumor models showed decreased expression of Slug. These results showed that HOTAIR may serve as a potential therapeutic target for GBM *in vivo*.

## DISCUSSION

Because EGFR activation is common in GBM, blocking the tyrosine kinase activity of EGFR has been the most attractive strategy for anti-GBM therapy [32]. Although kinase inhibitors (for example, erlotinib and gefitinib) and monoclonal antibodies (such as cetuximab and nimotuzumab) that target the EGFR pathway have been widely used, the outcome of GBM patients remains poor [33]. Targeting non-coding RNA could sensitize human glioma cell to anti-EGFR therapy [34]. To identify a better therapeutic target for this highly malignant tumor, we first reported the novel mechanism that long non-coding RNA HOTAIR regulates cell cycle progression and invasion through an NLK/β-catenin axis in GBM samples with different EGFR statuses.

The HOTAIR gene is located within the HOXC gene cluster on chromosome 12 and encodes a 2.2 kb lincRNA molecule that repressed transcription in the more distal HOXD locus in foreskin fibroblasts [35]. Extensive functional studies have indicated that overexpression of HOTAIR occurs in the majority of human solid tumors; strongly suggesting that HOTAIR promotes cancer progression [9–16]. HOTAIR knockdown induced G0/G1 cell cycle arrest in lung adenocarcinoma cells by increasing p21 expression and inhibited gastric cancer cell invasion by inhibiting HER2 [36, 37]. In the present study, the survival rate of GBM patients expressing high levels of HOTAIR was significantly shorter than that of GBM patients expressing low levels of HOTAIR. Moreover, we screened a large number of differentially expressed genes by analyzing different genome atlas databases and our RNA sequencing database in HOTAIR-depleted U87 GBM cells. Among these differentially expressed genes, NLK is known to be an important modulator of the β-catenin pathway by affecting the binding of the β-catenin/TCF/LEF complex to its target DNA sequence by phosphorylating LEF1 [21, 38, 39]. Notably, the expression level of β-catenin (or its phosphorylated form) and its transcriptional activity were dramatically inhibited by the elevated NLK level in U87 and U87vIII cells. CHIP-PCR indicated that HOTAIR-mediated H3K27 trimethylation was responsible for decreased NLK expression, which contributed to a stable β-catenin level in GBM. According to previous knowledge, Methylguanine Methyl Transferase (MGMT) and ALDH1A3 promoter methylation, IDH1 mutation were important prognosis predictors to GBM. Interestingly, we found that MGMT promoter methylation status was statistically different between HOTAIR high and low expressed GBM samples and these findings remaindered that HOTAIR might involved in GBM DNA methylation patterns [40]. Thus, our data suggested that cell cycle arrest and attenuation of invasion in GBM cells could be explained by HOTAIR depletion and subsequent inhibition of β-catenin (see Supplementary Figure S2A and S2B).

Anti-epigenetic therapy of Wnt/β-catenin and EGFR pathway was consider to be mostly prevail for therapeutic purposes in GBM [41]. Many factors were reported to regulate Wnt/β-catenin pathway, including non-coding RNAs, DKK1, SFRP1, WIF1 and etc [42]. HOTAIR interacted with the PRC2 and LSD1 complexes through a different domain than what was required for its robust epigenetic regulatory function [10–13]. Importantly, we addressed which of the two domains of HOTAIR affected the NLK/β-catenin axis. To answer this question, DZNEP and 2PCPA were used to inhibit the PRC2 complex and the LSD1 complex separately; the aim of using this method was to study the function of the HOTAIR 5′ and 3′ domains. The U87 and U87vIII cells treated with the EZH2 inhibitor displayed similar phenotypic changes to HOTAIR-depleted cells. To sufficiently demonstrate this observation, we introduced the 5′ and 3′ domains of HOTAIR into astrocytoma cells, which were obtained from a grade II astrocytoma patient, using a lentivirus system to compensate HOTAIR expression. Interestingly, comparing with the 3′ domain of HOTAIR, cells treated with the 5′ domain of HOTAIR showed decreased expression of NLK, RB and E-cadherin and increased expression of β-catenin, PKM2, CyclinD1, N-cadherin and Slug. EZH2 is the core component of the PRC2 complex, which mediates the transcriptional repression of pro-differentiation genes in both normal and tumor cells [43]. EZH2 is highly expressed in malignant glioma, and its expression positively correlates with malignancy and poor patient prognosis [44–46]. Noncoding RNAs were proven involved in regulating EZH2, including miRNA and lncRNA [47]. Although the function of EZH2 is still unclear, recent research revealed that phosphorylation of EZH2 at serine 21 (pS21 EZH2) by AKT facilitates STAT3 methylation by EZH2 and enhances STAT3 activity, which is necessary to maintain GBM stem-like cell self-renewal and tumor malignancy [48]. Based on our data, we deduced that the predominant function of HOTAIR in regulating GBM growth is the recruitment of the PRC2 complex to mediate target gene silencing by the 5′ domain of HOTAIR.

In summary, our integrated analyses have identified that HOTAIR is a negative prognostic factor for GBM patients. We validated that knockdown of HOTAIR inhibited cell cycle progression and invasion of GBM cells. These effects were independent of EGFR activation status, highlighting the potential of HOTAIR as a candidate therapeutic target in GBM. Therefore, inhibiting HOTAIR activity with molecular inhibitors may lead to a favorable clinical outcome in patients with GBM.

## MATERIALS AND METHODS

### Datasets of glioma samples

MRNA expression datasets and the corresponding clinical information were downloaded from websites as follows: CGGA1 and CGGA2 (http://www.cgga.org.cn), TCGA (http://cancergenome.nih.gov), REMBRANDT (http://caintegrator.nci.nih.gov/rembrandt/), TTseq (http://www.ncbi.nlm.nih.gov/geo/query/acc.cgi?acc=GSE48865) and GSE4290 (http://www.ncbi.nlm.nih.gov/geo/query/acc.cgi?acc=gse4290).

### Immunohistochemistry for MGMT, Ki-67, EGFR and PTEN

Immunoperoxidase staining for MGMT, Ki-67, EGFR and MGMT (Santa Cruz Biotechnology) of 109 primary gliomblastoma samples from CGGA1 dataset was performed on formalin-fixed, paraffin-embedded tissue sections, according to the manufacturer's instructions. Each stained slide was jointly scored by 2 pathologists blinded to the clinical information. For statistical analysis, high expression (H) of EGFR and MGMT was defined as > 30% of positive-stained cells in the tumor (L, < 30%; ND, not determined because of sample limitation). The p53 and Ki-67 protein accumulation was defined as strong nuclear staining in at least 30% of the tumor cells. Two blinded pathologists independently evaluated the slides. In case of a discrepancy, the 2 observers simultaneously reviewed the slides to achieve a consensus.

### Cell lines and culture conditions

The human GBM cell lines U87, U87vIII the breast cancer cell line MCF-7; and a glioma primary cultured cell line (astrocytoma) were used for experiments. U87vlll cell carries a truncated mutant EGFR gene, which can be activated without EGF stimulation consistently [31]. U87 and U87vlll GBM cells can represent GBM cell with or without EGFR activation. All cells were cultured in DMEM supplemented with 10% FBS, see details in the Supplementary Materials and Methods.

### Drug treatments

We employed DZNEP (Merck Millipore) to inhibit the PRC2 complex (EZH2 is the catalyzed subunit) activity and 2PCPA (Merck Millipore) to inhibit LSD1 complex activity. For EZH2 cells were treated with 5 μmol/L DZNEP dissolved in dimethyl sulfoxide (DMSO, Sigma-Aldrich) or 2PCPA dissolved in DDW for 48 hours.

### Lentiviral infection

Lentiviruses containing a HOTAIR inhibitor sequence (Lenti-HOTAIR si) or negative control (Lenti-NC) sequence were obtained from Shanghai Genepharma, China. Lentiviruses containing the HOTAIR 3′ or 5′ domain were obtained from Shanghai Genechem, China. See details in the Supplementary Materials and Methods.

Additional experimental procedures for RNA extraction and whole-genome gene profiling, qRT-PCR [49], mRNA sequencing and analysis, cell experiments *in vitro* [49–51] co-immunoprecipitation [51], chromatin immunoprecipitation [52], Western blots [49], Immunofluorescence staining [49], animal experiments and histopathology [48] experiments are described in the Supplementary Materials and Methods.

### Statistical analysis

The statistical significance of differences between different groups was determined using Student's *t*-test. Gene profiling data were analyzed using either BRB-Array Tools44 or Gene Pattern software. Kaplan-Meier analysis and log-rank tests were used to evaluate the prognostic significance of HOTAIR expression level for patient survival. The Cox proportional hazards regression model was also used to evaluate independent prognostic factors for GBM samples with different levels of HOTAIR expression. One-way ANOVA was used to test for differences among at least 3 groups, and a least significant difference post-hoc test was used to obtain individual *P* values followed by ANOVA. The *t*-test was used to determine differences between 2 groups. *P* values < 0.05 were regarded as significant.

## SUPPLEMENTAL FIGURES AND TABLES



## References

[1] Van den Bent M, Chinot OL, Cairncross JG (2003). Recent developments in the molecular characterization and treatment of oligodendroglial tumors. Neuro Oncol.

[2] Norden AD, Drappatz J, Wen PY (2009). Antiangiogenic therapies for high-grade glioma. Nat Rev Neurol.

[3] Arteaga CL (2002). Epidermal growth factor receptor dependence in human tumors: more than just expression?. Oncologist.

[4] Heimberger AB, Hlatky R, Suki D, Yang D, Weinberg J, Gilbert M, Sawaya R, Aldape K (2005). Prognostic effect of epidermal growth factor receptor and EGFRvIII in glioblastoma multiforme patients. Clin Cancer Res.

[5] Mellinghoff IK, Wang MY, Vivanco I, Haas-Kogan DA, Zhu S, Dia EQ, Lu KV, Yoshimoto K, Huang JH, Chute DJ, Riggs BL, Horvath S, Liau LM (2005). Molecular determinants of the response of glioblastomas to EGFR kinase inhibitors. N Engl J Med.

[6] Gan HK, Kaye AH, Luwor RB (2009). The EGFRvIII variant in glioblastoma multiforme. J Clin Neurosci.

[7] Reardon DA, Wen PY (2006). Therapeutic advances in the treatment of glioblastoma: rationale and potential role of targeted agents. Oncologist.

[8] Mattick JS (2004). RNA regulation: a new genetics?. Nat Rev Genet.

[9] Auffinger B, Thaci B, Ahmed A, Ulasov I, Lesniak MS (2013). MicroRNA targeting as a therapeutic strategy against glioma. Curr Mol Med.

[10] Khalil AM, Guttman M, Huarte M, Garber M, Raj A, Rivea Morales D, Thomas K, Presser A, Bernstein BE, van Oudenaarden A, Regev A, Lander ES, Rinn JL (2009). Many human large intergenic noncoding RNAs associate with chromatin-modifying complexes and affect gene expression. Proc Natl Acad Sci USA.

[11] Gupta RA, Shah N, Wang KC, Kim J, Horlings HM, Wong DJ, Tsai MC, Hung T, Argani P, Rinn JL, Wang Y, Brzoska P, Kong B (2010). Long non-coding RNA HOTAIR reprograms chromatin state to promote cancer metastasis. Nature.

[12] Tsai MC, Manor O, Wan Y, Mosammaparast N, Wang JK, Lan F, Shi Y, Segal E, Chang HY (2010). Long noncoding RNA as modular scaffold of histone modification complexes. Science.

[13] Ouyang Z, Zheng GX, Chang HY (2010). Noncoding RNA landmarks of pluripotency and reprogramming. Cell Stem Cell.

[14] Yang Z, Zhou L, Wu LM, Lai MC, Xie HY, Zhang F, Zheng SS (2011). Over-expression of long noncoding RNA HOTAIR predicts tumor recurrence in hepatocellular carcinoma patients following liver transplantation. Ann Surg Oncol.

[15] Kogo R, Shimamura T, Mimori K, Kawahara K, Imoto S, Sudo T, Tanaka F, Shibata K, Suzuki A, Komune S, Miyano S, Mori M (2011). Long noncoding RNA HOTAIR regulates polycomb-dependent chromatin modification and is associated with poor prognosis in colorectal cancers. Cancer Res.

[16] Zhang JX, Han L, Bao ZS, Wang YY, Chen LY, Yan W, Yu SZ, Pu PY, Liu N, You YP, Jiang T, Kang CS (2013). Chinese Glioma Cooperative Group. HOTAIR, a cell cycle- associated long noncoding RNA and a strong predictor of survival, is preferentially expressed in classical and mesenchymal glioma. Neuro Oncol.

[17] Shi Z, Qian X, Li L, Zhang J, Zhu S, Zhu J, Chen L, Zhang K, Han L, Yu S, Pu P, Jiang T, Kang C (2012). Nuclear translocation of β-catenin is essential for glioma cell survival. J Neuroimmune Pharmacol.

[18] Kaur N, Chettiar S, Rathod S, Rath P, Muzumdar D, Shaikh ML, Shiras A (2013). Wnt3a mediated activation of Wnt/β-catenin signaling promotes tumor progression in glioblastoma. Mol Cell Neurosci.

[19] Kim KH, Seol HJ, Kim EH, Rheey J, Jin HJ, Lee Y, Joo KM, Lee J, Nam DH (2013). Wnt/β-catenin signaling is a key downstream mediator of MET signaling in glioblastoma stem cells. Neuro Oncol.

[20] Zhang K, Zhang J, Han L, Pu P, Kang C (2012). Wnt/beta-catenin signaling in glioma. J Neuroimmune Pharmacol.

[21] Ishitani T, Ninomiya-Tsuji J, Nagai S, Nishita M, Meneghini M, Barker N, Waterman M, Bowerman B, Clevers H, Shibuya H, Matsumoto K (1999). The TAK1-NLK-MAPK-related pathway antagonizes signalling between beta-catenin and transcription factor TCF. Nature.

[22] Cancer Genome Atlas Research Network (2008). Comprehensive genomic characterization defines human glioblastoma genes and core pathways. Nature.

[23] Wang Z, Bao Z, Yan W, You G, Wang Y, Li X, Zhang W (2013). Isocitrate dehydrogenase 1 mutation-specific microRNA signature predicts favorable prognosis in glioblastoma patients with IDH1 wild type. J Exp Clin Cancer Res.

[24] Madhavan S, Zunklusen JC, Kotliarov Y, Sahni H, Fine HA, Buetow K (2009). Rembrandt: helping personalized medicine become a reality through integrative translational research. Mol Cancer Res.

[25] Bao ZS, Chen HM, Yang MY, Zhang CB, Yu K, Ye WL, Hu BQ, Yan W, Zhang W, Akers J, Ramakrishnan V, Li J, Carter B, Liu YW, Hu HM, Wang Z, Li MY, Yao K, Qiu XG, Kang CS, You YP, Fan XL, Song WS, Li RQ, Su XD, Chen CC, Jiang T (2014). RNA-seq of 272 gliomas revealed a novel, recurrent PTPRZ1-MET fusion transcript in secondary glioblastomas. Genome Res.

[26] Sun L, Hui AM, Su Q, Vortmeyer A, Kotliarov Y, Pastorino S (2006). Neuronal and glioma-derived stem cell factor induces angiogenesis within the brain. Cancer Cell.

[27] Canel M, Serrels A, Frame MC, Brunton VG (2013). E-cadherin-integrin crosstalk in cancer invasion and metastasis. J Cell Sci.

[28] Bae GY, Choi SJ, Lee JS, Jo J, Lee J, Kim J, Cha HJ (2013). Loss of E-cadherin activates EGFR-MEK/ERK signaling, which promotes invasion via the ZEB1/MMP2 axis in non-small cell lung cancer. Oncotarget.

[29] Wang Y, Lin Z, Sun L, Fan S, Huang Z, Zhang D, Yang Z, Li J, Chen W (2014). Akt/Ezrin Tyr353/NF-κB pathway regulates EGF-induced EMT and metastasis in tongue squamous cell carcinoma. Br J Cancer.

[30] Vesuna F, van Diest P, Chen JH, Raman V (2008). Twist is a transcriptional repressor of E-cadherin gene expression in breast cancer. Biochem Biophys Res Commun.

[31] Zheng Q, Han L, Dong Y, Tian J, Huang W, Liu Z, Jia X, Jiang T, Zhang J, Li X, Kang C, Ren H (2014). JAK2/STAT3 targeted therapy suppresses tumor invasion via disruption of the EGFRvIII/JAK2/STAT3 axis and associated focal adhesion in EGFRvIII-expressing glioblastoma. Neuro Oncol.

[32] Wang Y, Jiang T (2013). Understanding high grade glioma: molecular mechanism, therapy and comprehensive management. Cancer Lett.

[33] Kalman B, Szep E, Garzuly F, Post DE (2013). Epidermal growth factor receptor as a therapeutic target in glioblastoma. Neuromolecular Med.

[34] Zhang KL, Han L, Chen LY, Shi ZD, Yang M, Ren Y, Chen LC, Zhang JX, Pu PY, Kang CS (2014). Blockage of a miR-21/EGFR regulatory feedback loop augments anti-EGFR therapy in glioblastomas. Cancer Lett.

[35] Wang KC, Yang YW, Liu B, Sanyal A, Corces-Zimmerman R, Chen Y, Lajoie BR, Protacio A, Flynn RA, Gupta RA, Wysocka J, Lei M, Dekker J (2011). A long noncoding RNA maintains active chromatin to coordinate homeotic gene expression. Nature.

[36] Liu Z, Sun M, Lu K, Liu J, Zhang M, Wu W, De W, Wang Z, Wang R (2013). The long noncoding RNA HOTAIR contributes to cisplatin resistance of human lung adenocarcinoma cells via downregualtion of p21(WAF1/CIP1) expression. PLoS One.

[37] Liu XH, Sun M, Nie FQ, Ge YB, Zhang EB, Yin DD, Kong R, Xia R, Lu KH, Li JH, De W, Wang KM, Wang ZX (2014). Lnc RNA HOTAIR functions as a competing endogenous RNA to regulate HER2 expression by sponging miR-331–3p in gastric cancer. Mol Cancer.

[38] Ishitani T, Ninomiya-Tsuji J, Matsumoto K (2003). Regulation of Lymphoid Enhancer Factor 1 T-Cell Factor by Mitogen-Activated Protein Kinase-Related Nemo-Like Kinase-Dependent Phosphorylation in Wnt β-Catenin Signaling. Mol Cell Biol.

[39] Smit L, Baas A, Kuipers J, Korswagen H, van de Wetering M, Clevers H (2004). Wnt activates the Tak1/Nemo-like kinase pathway. J Biol Chem.

[40] Zhang W, Yan W, You G, Bao Z, Wang Y, Liu Y, You Y, Jiang T (2013). Genome-wide DNA methylation profiling identifies ALDH1A3 promoter methylation as a prognostic predictor in G-CIMP-primary glioblastoma. Cancer Lett.

[41] Paul I, Bhattacharya S, Chatterjee A, Ghosh MK (2013). Current Understanding on EGFR and Wnt/β-Catenin Signaling in Glioma and Their Possible Crosstalk. Genes Cancer.

[42] Foltz G, Yoon JG, Lee H, Ma L, Tian Q, Hood L, Madan A (2010). Epigenetic regulation of wnt pathway antagonists in human glioblastoma multiforme. Genes Cancer.

[43] Kleer CG, Cao Q, Varambally S, Shen R, Ota I, Tomlins SA, Ghosh D, Sewalt RG, Otte AP, Hayes DF, Sabel MS, Livant D, Weiss SJ (2003). EZH2 is a marker of aggressive breast cancer and promotes neoplastic transformation of breast epithelial cells. Proc Natl Acad Sci U S A.

[44] Cao R, Wang L, Wang H, Xia L, Erdjument-Bromage H, Tempst P, Jones RS, Zhang Y (2002). Role of histone H3 lysine 27 methylation in Polycomb-group silencing. Science.

[45] Crea F, Hurt EM, Farrar WL (2010). Clinical significance of Polycomb gene expression in brain tumors. Mol Cancer.

[46] Wu Z, Wang Q, Wang L, Li G, Liu H, Fan F, Li Z, Li Y, Tu Y (2013). Combined aberrant expression of Bmi1 and EZH2 is predictive of poor prognosis in glioma patients. J Neurol Sci.

[47] Smits M, Nilsson J, Mir SE, van der Stoop PM, Hulleman E, Niers JM, de Witt Hamer PC, Marquez VE, Cloos J, Krichevsky AM, Noske DP, Tannous BA, Würdinger T (2010). miR-101 is down-regulated in glioblastoma resulting in EZH2-induced proliferation, migration, and angiogenesis. Oncotarget.

[48] Kim E, Kim M, Woo DH, Shin Y, Shin J, Chang N, Oh YT, Kim H, Rheey J, Nakano I, Lee C, Joo KM, Rich JN (2013). Phosphorylation of EZH2 activates STAT3 signaling via STAT3 methylation and promotes tumorigenicity of glioblastoma stem-like cells. Cancer Cell.

[49] Zhou X, Ren Y, Moore L, Mei M, You Y, Xu P, Wang B, Wang G, Jia Z, Pu P, Zhang W, Kang C (2010). Downregulation of miR-21 inhibits EGFR pathway and suppresses the growth of human glioblastoma cells independent of PTEN status. Lab Invest.

[50] Zhou X, Ren Y, Liu A, Han L, Zhang K, Li S, Li P, Li P, Kang C, Wang X, Zhang L (2014). STAT3 inhibitor WP1066 attenuates miRNA-21 to suppress human oral squamous cell carcinoma growth *in vitro* and *in vivo*. Oncol Rep.

[51] Zhang KL, Zhou X, Han L, Chen LY, Chen LC, Shi ZD, Yang M, Ren Y, Yang JX, Frank TS, Zhang CB, Zhang JX, Pu PY (2014). MicroRNA-566 activates EGFR signaling and its inhibition sensitizes glioblastoma cells to nimotuzumab. Mol Cancer.

[52] Han L, Yue X, Zhou X, Lan FM, You G, Zhang W, Zhang KL, Zhang CZ, Cheng JQ, Yu SZ, Pu PY, Jiang T, Kang CS (2012). MicroRNA-21 expression is regulated by β-catenin/STAT3 pathway and promotes glioma cell invasion by direct targeting RECK. CNS Neurosci Ther.

