# Supplementary material for: HOTAIR is a therapeutic target in glioblastoma
Source: Oncotarget. 2015 Mar 21;6(10):8353–65. doi: 10.18632/oncotarget.3229 (PMC4480757; doi:10.18632/oncotarget.3229)
Supplement: Supplementary file 1 [file oncotarget-06-8353-s001.pdf]

SUPPLEMENTARY MATERIALS AND METHODS

Cell lines and culture conditions

The human GBM cell lines U87 and the breast cancer cell line MCF-7 were obtained from Institute of Biochemistry and Cell Biology, Chinese Academy of Science (Shanghai, China). The U87vIII GBM cell line, which carries a mutant EGFR, was kindly provided by Prof. Xia Li of the College of Bioinformatics Science and Technology, Harbin Medical University (Harbin, China). A primary-cultured glioma cell line (astrocytoma) was derived from the resected tumor of a 57-year-old oligodendroglioma patient. All the cell lines were grown and maintained in Dulbecco's modified Eagle's medium nutrient mixture (Gibco, USA) supplemented with 10% fetal bovine serum, 2 mM glutamine (Sigma, USA), 100 units of penicillin/ml (Sigma), and 100 mg of streptomycin/ml (Sigma) at 37°C with 5% CO_2.

Lentiviral infection

Lentiviruses containing a HOTAIR inhibitor sequence (Lenti-HOTAIR si) or negative control (Lenti-NC) sequence were obtained from Genepharma (Shanghai, China). Transfection was performed according to the manufacturer's instructions. The human glioma cell lines U87 and U87vIII and the breast cancer cell line MCF-7 were infected with lentiviruses at 70% confluence. The infected cells were harvested for further use after 48 hours.

qRT-PCR

Total RNA was extracted using TRIzol reagent (Life Technologies). To detect HOTAIR, quantitative reverse transcription-polymerase chain reaction (qRT-PCR) was performed using a Reverse Transcription System (Promega) according to the manufacturer's instructions. All HOTAIR expression data were normalized to GAPDH from the same sample. For primers for HOTAIR and GAPDH, see Supplementary Table S1.

mRNA sequencing and analysis

The total RNA extracted from U87 cells infected with Lenti-HOTAIR si or NC virus was first treated with DNase I to degrade any possible contaminating DNA. The mRNA was then enriched using oligo(dT) magnetic beads (for eukaryotes) or by removing rRNAs from the total RNA (for prokaryotes). Then, mRNA was then mixed with fragmentation buffer and fragmented into short fragments (approximately 200 bp). The first strand of cDNA was synthesized using random hexamer primers. Buffer, dNTPs, RNase H and DNA polymerase I were added to synthesize the second strand. The double-stranded cDNA was purified with magnetic beads. End reparation and 3'-end single nucleotide A (adenine) addition were then performed. Finally, sequencing adaptors were ligated onto the fragments. The fragments were enriched by PCR amplification. During the QC step, an Agilent 2100 Bioanalyzer and ABI StepOnePlus Real-Time PCR System were used to analyze and quantify the sample library. The library products were then sequenced via an Illumina HiSeqTM 2000.

Single-end FASTQ sequence files consisting of 49 bp were generated for U87 cells infected with Lenti-HOTAIR si or NC virus. Each read was aligned with BWA software against Homo sapiens UCSC hg19 references. Uniquely mapped reads with mapping qualities larger than or equal to 20 were retrieved. Using uniquely mapped reads, read counts per exon were obtained, and the read counts for all exons within a gene were summarized for all genes in the UCSC hg19 refFlat table. For each sample, RPKM was calculated as the number of reads that mapped per kilobase of exon model per million of mapped reads for each gene. Differentially expressed genes between samples were determined using the R-package DEGseq with the method MARS (MA-plot-based method with random sampling model), fold change cutoff = 1.5, *P* value cutoff = 0.00001, and absolute change bigger than 50 reads.

Cell experiments *in vitro*

For cell cycle analysis by flow cytometry, NC and HOTAIR-depleted cells were harvested in the log phase of growth, washed with PBS and fixed with 90% ethanol overnight at 4–8°C. Cell nuclei were stained with propidium iodide for an additional 30 min. A total of 10,000 nuclei were examined in a FACS Calibur flow cytometer (Becton-Dickinson), and DNA histograms were analyzed using Modifit software.

For Transwell assays, Corning Transwell insert chambers (Corning, USA) and BD Matrigel Invasion Chambers (BD Biosciences) were used. The prepared cells were added to the chamber and incubated for 24 h at 37°C. Cells that invaded the lower chamber through the membrane were fixed with 20% methanol and stained with 0.1% crystal violet. The number of cells invading through the matrigel was counted using 3 randomly selected visual fields from the central and peripheral portions of the filter.

For wound healing assays, 10,000 GBM cells infected with Lenti-HOTAIR si or Lenti-NC were seeded into a 12-well plate. After 24 h, a straight scratch was made with a 200 µl pipette tip, and the wound was photographed under the microscope. After 48 h, cells were photographed under a DP-70 microscope (Olympus).

For soft agar assays, 1,000 GBM cells infected with Lenti-HOTAIR si or Lenti-NC were added to 3 ml of

DMEM with 0.3% agar (BD Bioscience) and layered onto 6 ml 0.5% agar beds in 60 mm dishes. Cells were treated with different drugs and cultured for 2 weeks, after which time the colonies were photographed. Colonies larger than 50 μm in diameter were counted as positive for growth using the DP-70 microscope (Olympus).

Co-Immunoprecipitation

For immunoprecipitation, cells were lysed in IP lysis buffer (Pierce, Rockford, USA). The cell lysates were then subjected to immunoprecipitation with 1–5 mg of antibodies and Protein A/G agarose beads (Pierce, Rockford, USA) overnight at 4°C with constant agitation. Control samples were incubated with agarose beads after immunoprecipitation with a control immunoglobulin. The immunoprecipitated complexes were then washed with wash buffer. The proteins were eluted, boiled and subjected to SDS-PAGE analysis.

Chromatin immunoprecipitation assay

U87 cells were harvested for chromatin immunoprecipitation (ChIP) using a Methyl-Histone H3K27 ChIP Kit (EpiQuik, USA) according to the manufacturer's protocols. Briefly, the cells were cross-linked *in vivo* using 1% formaldehyde for 10 min and incubated with lysis buffer for 10 min, and DNA was sheared by sonicating for 3 to 4 pulses of 10–12 sec. The cross-linked DNA was reversed by treatment with DNA release buffer containing proteinase K at 65°C for 15 min, and the purified DNA was diluted in 10–20 μl of elution buffer. The PCR primers for the NLK gene are shown in Supplementary Table S2.

Western blots and Immunofluorescence staining

Parental, lentivirus-infected, and DZNEP- or 2PCPA-treated cells were washed three times with pre-chilled phosphate-buffered saline (PBS). The cells were collected and then lysed with RIPA reagent (Solarbio Biotechnology). A total of 40 mg of lysates was subjected to SDS-PAGE on an 8% SDS-polyacrylamide gel. Separated proteins were transferred to PVDF membranes (Merck Millipore) and incubated with primary antibodies followed by incubation with an HRP-conjugated secondary antibody. The bands were detected using the Immobilon ECL system (Merck Millipore). The membranes were stripped and re-probed with a primary antibody against GAPDH (Santa Cruz Biotechnology).

For immunofluorescence staining, U87, U87vIII and MCF7 cells were seeded onto poly-L-lysine-treated coverslips (BD Biosciences). The cells were then infected with Lenti-HOTAIR si or Lenti-NC. After 48 h, the cells were fixed in 4% paraformaldehyde for 2 min. The cells were then washed 3 times in PBS and incubated in 5% bovine serum albumin (BSA) for 30 min at room temperature. Next, the cells were washed in PBS and incubated overnight at 4°C with primary antibodies. The cells were again washed in PBS, followed by incubation with a fluorescent secondary antibody for 1 h at 37°C. For F-actin staining, phalloidin (Life Technologies) was incubated with the cells for 15 min at room temperature. Nuclei were stained with DAPI solution for 5 min. Confocal images of the cells were acquired on an FV-1000 laser scanning confocal microscope (Olympus).

Orthotopic GBM Model and Management

Four-week-old Bagg albino (BALB)/c female nude mice were purchased from the Animal Center of the Cancer Institute at the Chinese Academy of Medical Science. All mice were bred at Compare Medicine Center, Tianjin Medical University. All experimental procedures were performed according to Tianjin Medical University Medical Research Used Animal Principle policies. To establish intracranial gliomas, 0.5×10^5 U87 and U87vIII cells were infected with Lenti-HOTAIR si or Lenti-NC virus and then implanted stereotactically. Mice were imaged for Fluc activity using bioluminescence imaging (Caliper IVIS Spectrum, USA) on days 7, 21, and 21.

Hematoxylin and eosin staining (HE) and immunohistochemistry analysis (IHC)

By the end of the observation period, all nude mice were sacrificed, and the mouse brains were prepared for further pathological examination. Paraffin-embedded tissue sections were used for HE staining and IHC analysis as previously described (21). Briefly, for IHC analysis, the sections were incubated with primary antibodies (1:100 dilutions) overnight at 4°C, followed by a biotin-labeled secondary antibody (1:100 dilutions) for 1 h at 37°C and then incubated with DAB (3,3'-diaminobenzidine) Substrate Solution (Zhongshan Bio Corp), counterstained with hematoxylin (Zhongshan Bio Corp) and visualized using a light microscope.

SUPPLEMENTARY FIGURES AND TABLES

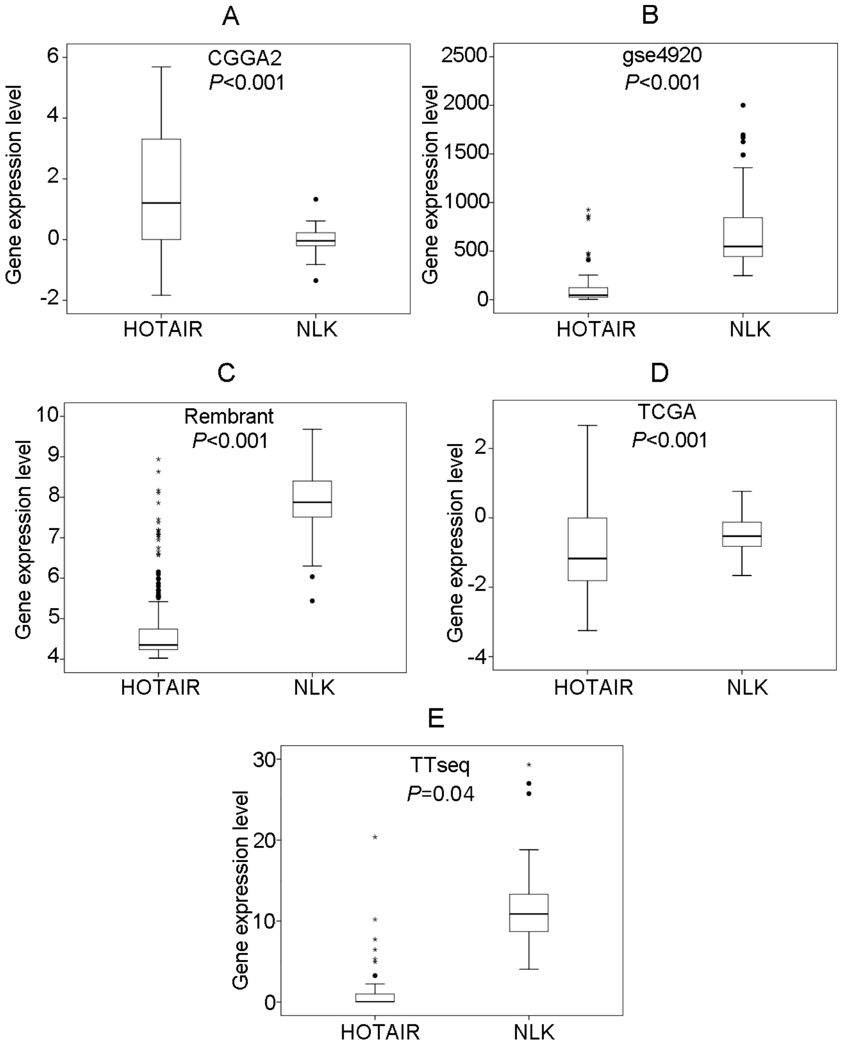

Supplementary Figure S1: HOTAIR expression was negative associated with NLK in public glioma atlas databases. (A) CGGA2, *P* < 0.001; (B) gse4920, *P* < 0.001; (C) Rembrant, *P* < 0.001; (D) TCGA, *P* < 0.001; (E) TTseq, *P* = 0.04).

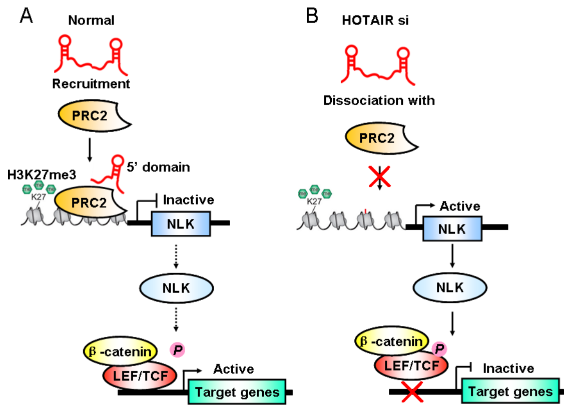

Supplementary Figure S2: The working model indicating that HOTAIR regulates β-catenin transcriptional activity by inhibiting NLK in GBM. (A) HOTAIR inhibited β-catenin transcriptional activity by inhibiting NLK. B: HOTAIR depletion increased β-catenin transcriptional activity.

Supplementary Table S1: The PCR primers for GADPH and HOTAIR

	Up stream	Down stream
HOTAIR	5′-ggtagaaaaagcaaccacgaagc-3′	5′-acataaacctctgtctgtgagtgcc-3′
GADPH	5′-ccgggaaactgtggcgtgatgg-3′	5′-aggtggaggagtgggtgtcgtgtt-3′

Supplementary Table S2: The PCR primers for NLK gene promoter and 5′UTR

	Up stream	Down stream
NLK primer 1	5′-ccattcagcctttcattttca-3′	5′-ccgataagcccatcgtatgt-3′
NLK Primer2	5′- tcatgccaggctatttaggg-3′	5′-tagcttgagccagggaggta-3′
NLK Primer3	5′- caggcagggaaatggttaaa-3′	5′-cccaggctgaatcatcaagt-3′
NLK Primer4	5′- ctgtgccactgcagtctagc-3′	5′-tcttggcaaaaagaggagga-3′
NLK Primer5	5′- cctccttccctggcttattc-3′	5′-ccaccaccctaaatggagaa-3′
